# Role of food in environmental transmission of *Helicobacter pylori*

**DOI:** 10.22088/cjim.8.3.146

**Published:** 2017

**Authors:** Mohammad Zamani, Amin Vahedi, Zahra Maghdouri, Javad Shokri-Shirvani

**Affiliations:** 1 Student Research Committee, Babol University of Medical Sciences, Babol, Iran.; 2 Cancer Research Center, Health Research Institute, Babol University of Medical Sciences, Babol, Iran.; 3 School of Medicine, Babol University of Medical Sciences, Babol, Iran.; 4 Department of Internal Medicine, Ayatollah Rohani Hospital, Babol University of Medical Sciences, Babol, Iran.

**Keywords:** *Helicobacter pylori*, Transmission, Food, Water, Reservoir

## Abstract

*Helicobacter pylori* (*H.pylori*) is a gram-negative bacterium that has infected more than half of the world's population. This pathogen colonizes the human gastric mucosa and is usually acquired during childhood. It is an important cause of peptic ulcers, chronic gastritis and stomach cancer. Among the risk factors for acquisition of *H. pylori* infection, poor socioeconomic status, poor sanitization and hygiene practices, and contaminated food and water, are the most significant ones. The main route of *H. pylori* transmission is still unknown. Studies show that *H.pylori* bacteria can spread directly from one person to the other, or indirectly from an infected person to the environment. Person to person transmission is divided into fecal-oral, gastric-oral, oral-oral, sexual routes. Presently, interpersonal pathways are more acceptable than environmental exposure routes. Literatures indicate the presence and survival of *H. pylori* in food samples, such as milk, vegetables and meat, and suggest these foods may play an important role in the environmental transmission of this pathogen. In addition, other studies report the presence of *H. pylori* in the gastric tissue of some animals (e.g. sheep and cow) and therefore, it is likely they participate in the food chain transmission as reservoirs besides human. Although there are findings which indicate the probable role of food products in the environmental transmission of *H. pylori*, there is still not enough direct evidence to confirm this and more studies are needed. However, attention to food contamination sources (unhygienic water) and controlling them may prevent transmission of pathogens associated with health.

*Helicobacter pylori* is a spiral-shaped gram-negative microaerophilic bacterium which is found in the human gastric mucosa. This pathogen was first isolated by Warren and Marshall (1) about 30 years ago. It was isolated from the human stomach but the principle mechanism by which it colonizes is still unclear (2, 3). Human stomach is currently the only known reservoir for this pathogen. The bacterial pathogen *H. pylori* infects about 50% of the human population around the world. Seroepidemiological studies of *H. pylori* show that the rate of infection regionally changes and it is lower in developed countries (about 30-40%) compared with developing countries (in some areas, >85%) (4-6). According to reports, its prevalence has declined in the world, which can be explained by the improvement of hygiene (7). This microorganism can be associated with the pathogenesis of some gastrointestinal diseases, such as chronic antral gastritis of type B, peptic ulcers, mucosa associated lymphoid tissue lymphoma and gastric adenocarcinoma (8-13). Furthermore, possible associations have been reported between *H. pylori* and a number of extragastric manifestations related to cardiovascular, dermatological, neurological, immunological, hematological, hepatobiliary, respiratory, and endocrine and metabolic disorders (14-17). 

On other hand, increasing antimicrobial resistance of *H. pylori* has increased concerns about treatment failure and lack of control of the important mentioned gastrointestinal diseases (18, 19). Several epidemiologic risk factors for acquisition of *H. pylori* infection have been highlighted and are summarized in figure 1 (4, 20, 21). Most of risk factors are related to poor living conditions and there is no difference in this respect between the developed and developing countries (22). There are inconsistent findings regarding the association between *H. pylori* infection and some factors, such as gender and lifestyle habits (e.g. smoking and alcohol drinking) (3, 23).

The principle mechanism by which *H. pylori* infection is transmitted to humans is still not exactly defined, however, person to person and environment to person transmissions are two potential options according to studies. Interpersonal transmission may occur via several pathways, including fecal-oral, gastric-oral, oral-oral, and sexual (24-26).

**Figure 1:**
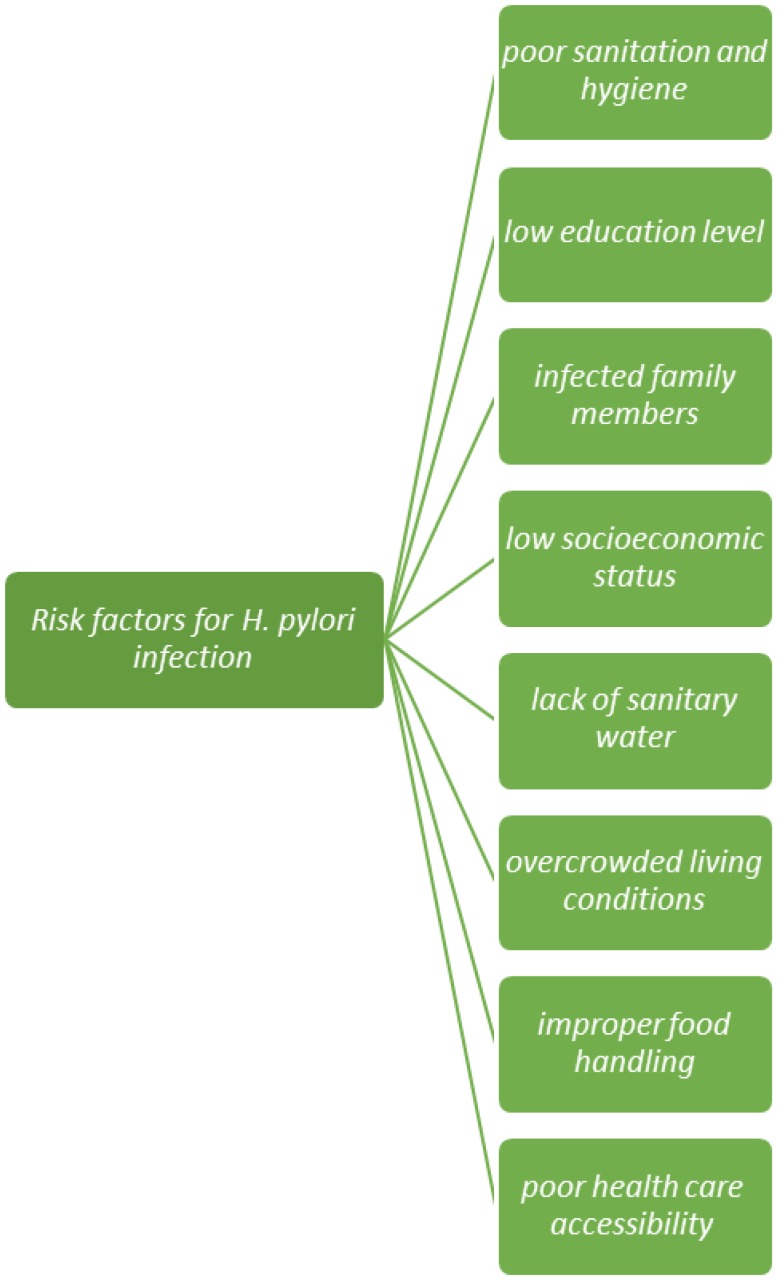
Main potential risk factors for *H. pylori* infection

**Evidence for the role of food in environmental** **transmission**

**A) Food as a route of transmission: **The hypothesis that food is a route of transmission of *H. pylori* is supported by epidemiologic studies that have observed a higher prevalence of *H. pylori* infection and a more rapid acquisition rate in areas with poor hygienic conditions (27-29). Altogether, the food-borne transmission of *H. pylori* can be attributed to two modes of person to person and environment to person. For instance, the infection might be transmitted by ingesting foods from street vendors (22, 30). This infection may occur directly by vendors (interpersonal) or indirectly by contaminated foods (environmental). It has also been proposed that saliva could be a source of* H. pylori *(25), so food soaked in contaminated saliva can transfer the agent. On the other hand, there is some evidence supporting the role of contaminated foods in the environmental transmission of *H. pylori*, which are expressed below.

**B) Detection of** ***H. pylori*** **in food samples.**

**Dairy products:** According to the literatures, animal source foods, and specifically raw milk, have been considered as the most likely source of human infection in food chain transmission, since *H. pylori *DNA has been isolated from animal milk samples (e.g. sheep and cow) (31, 32). Of course, some other surveys reported no isolation of the organism from milk samples, which might be explained by the geographical spread of *H. pylori *(33, 34). This failure can also be due to the viable but nonculturable (VBNC) state of *H. pylori.* This state occurs to withstand environmental stressful conditions and starvation (35-37). It has been discussed that the process by which milk is contaminated may be related to lack of attention to hygiene measures during milking, cooling and storage (38). Examples of studies which have indicated that *H. pylori* DNA is present in raw milk are summarized in table 1. In addition, limited studies are available concerning the occurrence of *H. pylori *in dairy products other than milk. For example, in the study by Mousavi et al., 30% of cheese, 15% of cream, 5% of butter and 27% of ice cream samples, which were all made from unpasteurized milk, were positive for *H. pylori *(39).

Previous published data revealed the isolation of *H. pylori *from gastric tissue of several animals, taking part in the human food chain, such as sheep and cow, and this led researchers to presume them as plausible reservoirs and sources of the infection (32, 40, 41). This evidence also can support the theory of zoonotic transmission of *H. pylori. *Some studies addressed the high prevalence of *H. pylori *among shepherds and stated that sheep may play a vital role in *H. pylori* transmission in these people and their family members and the infection may originate from these animal species (42-44). According to the above data, and also on the possible transmission via sheep milk, *H. pylori* infection can be considered as zoonosis.

**Table 1:** Studies evaluating the presence of H. pylori DNA in milk

**Type of milk**	**Samples** **No.**	**Gene**	**Diagnostic method**	**Percentage of ** **positive samples**	**Reference**
Sheep raw	58	*cagA*	PCR	6.9	(59)
Cow raw	75			13.3	
Goat raw	42			4.7	
Buffalo raw	20			15	
Camel raw	15			6.6	
Bovine bulk	135	*ureC*	PCR	14.1	(38)
Ovine bulk	90			12.2	
Caprine bulk	103			8.7	
Camel bulk	55			3.6	
Buffalo bulk	64			23.4	
Buffalo bulk	210	*ureC*	PCR	11.4	(60)
Goat raw	160	*glmM*	Nested PCR	25.6	(61)
Sheep raw	130			33	
Cow raw	110			50	
Cow raw	20	16S rRNA	FISH	20	(62)
Sheep raw	63	16 rRNA & *vacA*	PCR	60.3 (16S rRNA) 7.9 (both of them)	(40)
Sheep raw	51	16 rRNA & *vacA*	PCR	60.3 (16S rRNA) 9.8 (both of them)	(63)
Cow raw	25	*ureC*	PCR	16	(64)
Cow raw	18	*ureA*	Semi-nested-PCR	72	(65)
Cow pasteurized	20			55	
Cow raw	120	*vacA*	PCR	20.83	(53)
Goat raw	80			18.75	
Sheep raw	120			29.16	
Camel raw	50			10	
Buffalo raw	50			24	

**Vegetables** **:** Few reports have addressed the occurrence of *H. pylori* in vegetables. For instance, in Iran, Atapoor et al. collected 460 vegetable and salad samples from supermarkets and grocery stores and examined them by culture and PCR. *H. pylori* was detected in 9.56% of samples by the culture technique, whereas PCR results showed that 10.86% of samples were positive (45). Also, Yahaghi et al. examined 380 mixed vegetable and 50 salad samples and reported that 13.68% of vegetable samples and 14% of salad samples were contaminated with *H. pylori *(46). Besides, Goodman et al. evaluated the prevalence of *H. pylori* infection in a rural community and reported that persons who are consumer of raw vegetables had more potential to be infected (47). 

These results reveal that vegetables may be likely sources of *H. pylori *and can play a significant role in the transmission of *H. pylori* to humans. Studies indicate that raw vegetables may become contaminated by irrigation water or unpurified water source used through washing (47, 48). At any rate, careful and adequate washing of raw vegetables may decrease the incidence of such contamination events (45).

**Yeasts:** Studies show that *H. pylori* can be found inside yeasts for example *Candida* spp. Iranian researchers in a number of studies, presented evidence for the existence of non-culturable *H. pylori *in the vacuole of *Candida* spp. from food products, such as breads, banana inner skin, yogurt, quince jam and grape juice (49, 50). On the other hand, yeasts can resist stressful conditions, for example high temperature, acidic pH and high sanitization (51). In conclusion, foodborne yeasts, such as *Candida* spp., which are often found in foods (e.g. raw milk), water and various human organs such as the oral cavity and the gastrointestinal and genitourinary tracts of humans, can act as a protector and reservoir of *H. pylori* in natural environments (49, 52).

**Other food products:** Saeidi et al. identified *H. pylori *in meat samples of cow (25%), sheep (37%), camel (14%), buffalo (28%) and goat (22%) (53). In their article, Hemmatinezhad et al. declared that 13.45% of ready-to-eat food samples, including cream-candy, traditional bread, salami, soup, restaurant salad, hamburger, sausage, falafel, fruit salad, chicken nugget and potato salad, were contaminated with *H. pylori *(54).

**C) Survival of** ***H. pylori*** **in foods**

**Food samples:** Some published data reveal that *H. pylori* can survive for short periods in artificially contaminated food products, such as milk, vegetables and meat, which are shown in table 2.

**Table 2:** Studies evaluating the survival of H. pylori* in inoculated foods

**Reference**	**Results**	**Type of food**
(66)	Recovered up to 5 days from pasteurized skim milk at 4 °C.	Milk
(67)	Survived in fresh milk without preservatives for up to 10 days at 4 °C and about 3 days at 25 °C.	
(68)	Survived for 9 days in pasteurized milk and 12 days in UHT milk at 4 ºC.	
(69)	Survived until 6 days in cooled milk, 3 to 4 days in milk at room temperature or 37 °C.	
(56)	Survived for 7 days at 22 °C.	Sucuk (Turkish fermented sausage)
(70)	Survived until 6 days at room temperature.	Spinach
(71)	Survived for 7 days in irradiated & 3 days in autoclaved ground beef at 37 °C.	Irradiated (10 kGy) & autoclaved ground beef
(67)	Survived at 4 °C until 2 days in lettuce & raw chicken, 5 days in tofu & not retrieved from yogurt sample.	Fresh leaf lettuce, raw chicken, tofu & plain yogurt
(72)	Survived at 8 ºC for up to 72 h in sanitized carrots & lettuce, until 96 h in sterilized carrots, up to 120 h in raw carrots.	Carrots & lettuce
(69)	Survived for 24 h in kefir, 10 h in curd cheese & 3 h in in yogurt at 37 °C, survived until 72 h in these three food products at 7 °C. Survived for 48 h in chicken at room temperature.	Kefir, curd cheese, yogurt & chicken

* The diagnostic method for *H. pylori* was culture for all above articles.

**Survival conditions:** Findings indicate that* H. pylori* is able to survive in water and milk, fresh fruits and vegetables, fresh meat (poultry, fish and red meat), at temperatures below 30°C (29,55). Besides, any food product presenting pH ranging from 4.9 to 6.0 and water activity higher than 0.97, can help this bacterium to have more survival potential (45). Besides, researchers found that *H. pylori*’s ability to survive in an acidic pH environment is dependently correlated with urea. Survival of *H. pylori* may be prolonged in milk due to the presence of urea in this food product (56). Based on the previously published documents, *H. pylori* does not seem to grow in most foods, but if kept refrigerated in stores, this microorganism can survive in low-acid and high-moisture settings for a long time (55). In addition, the frequent inability of sanitation schemes in clearing pathogens on raw vegetables and fruits has been predominantly ascribed to the inability of active components in treatment solutions to reach microbial cell sites (57, 58).

**Conclusion **Several studies indicate that this bacterium could be present in raw food products, such as milk and ready-to-eat foods like vegetables, and suggest that consumption of such foods maybe constitute a source of *H. pylori* infection for humans. Reports also show that some animals, like cow and sheep, could act as reservoirs of this pathogen, besides humans. Confirmation of the presence of *H. pylori* in foods is mainly based on indirect results that are too few to ascribe a definite foodborne role of *H. pylori* transmission. Consequently, it is needed that more epidemiological and experimental studies be performed to corroborate this hypothesis. Finding of new food sources and reservoirs of *H. pylori* can change or improve our knowledge in the future.
